# Optimization of Extraction Conditions of Bioactive Compounds From Kurdistan Species Urtica dioica

**DOI:** 10.7759/cureus.61146

**Published:** 2024-05-26

**Authors:** Mary H Haido, Alhan H Matti, Sherzad M Taher

**Affiliations:** 1 Pharmaceutics, University of Duhok, Duhok, IRQ; 2 Basic Sciences, University of Duhok, Duhok, IRQ

**Keywords:** non-conventional extraction techniques, conventional extraction methods, bioactive compounds, extraction conditions, urtica dioica

## Abstract

Introduction: Phytotherapeutics derived from medicinal plants treat various illnesses, including viral infections such as SARS, MERS, and SARSCoV-2, as well as bacterial and fungal diseases. It highlights ongoing research into the chemical compositions of plant components for developing new drugs, with a particular emphasis on anti-cytotoxic agents for anticancer drugs. Traditional extraction methods have limitations, leading to the exploration of environmentally friendly technologies such as ultrasound-assisted, supercritical fluid, microwave-assisted, and accelerated solvent extraction. The paragraph concludes by stating the aim of a specific study to optimize extraction conditions of bioactive compounds from *Urtica dioica* in Kurdistan, comparing conventional and non-conventional extraction methods, solvents, and extraction times.

Materials and methods: The study was conducted between June 2022 and August 2022, fresh leaves and stems of *U. dioica *plant were collected and sequentially underwent four extraction methods (maceration, Soxhlet, ultrasound-assisted extraction (UAE), and microwave-assisted extraction (MAE) by using petroleum ether, chloroform, ethanol, and distilled-water as solvents.

Results: The results highlighted significant variations in the yields of bioactive compounds based on the extraction method, solvent type, and duration. Among conventional methods, Soxhlet was the most powerful method and had the most extraction yields, while maceration had the lowest yields. The modern techniques surpassed the conventional methods by producing high extraction yields within a shorter time (a few minutes) and using a lesser amount of solvent. Consequently, UAE and MAE emerge as the most efficient techniques. Hence, MAE effectively produced the highest extraction yields and is considered the preferred technique. The choice of solvents significantly influenced the extraction yields, with ethanol consistently emerging as an effective solvent across various extraction methods. In contrast, petroleum ether demonstrated the lowest efficacy as a solvent. Furthermore, the results unveiled the impact of extraction time on yields, indicating a correlation between increased time and extraction yield in certain cases.

Conclusion: Extraction is a very critical step in the study of medicinal plants. The amount of extracted compounds is significantly affected by the extraction method, solvent, and time. Ethanol stands out as the most effective solvent, producing the highest yields of bioactive compounds, while petroleum ether yields the least. Additionally, extraction yield shows a direct relation with extraction time. Soxhlet being the most powerful among conventional methods and maceration yields the least. Modern techniques, particularly UAE and MAE, surpass conventional methods by achieving high yields in shorter times with less solvent. MAE, in particular, offers advantages such as shortened extraction time, increased efficiency, reduced labor, and enhanced selectivity, making it the preferred method for extracting bioactive compounds from aerial parts *U. dioica*.

## Introduction

Throughout history, plants have served as a vital source of therapeutic agents for human use. The World Health Organization (WHO) verified that the health demands of about 80% of the world’s population rely on herbal medicines and that approximately 20,000 plants are utilized for therapeutic purposes [1]. Numerous bioactive components, such as phenolics, alkaloids, saponins, terpenes, and lipids, can be found in plants and are very important. Active compounds are dispersed within plant tissues and cells, and their characteristics and concentration are influenced by factors such as plant species, the specific part used, maturity stage, extraction techniques, and other variables. Extraction serves as the initial step in plant-based drug research. In this crucial first step, the successful extraction of chemical components from plant material is essential for their identification and subsequent separation in the succeeding stages of the process. Moreover, the extraction of bioactive compounds for various industries, including pharmaceuticals, food, and chemicals, emphasizes the necessity for employing the most suitable and standardized extraction methods [2,3].

*Urtica dioica *L. (stinging nettle, common nettle) belongs to the family Urticaceae and is a perennial herb with a global distribution, and it is found worldwide, excluding Antarctica and certain tropical regions [4]. It is a very well-known plant having a wide historical background use of stems, leaves, and roots [5]. The plant encompasses a diverse array of phytochemicals, including phenolic compounds, fatty acids, sterols, alkaloids, flavonoids, terpenoids, and lignans, and scientific reports confirmed the presence of flavonoids, coumarins, phenolcarboxylic acids, sterols, carotenoids, amino acids, vitamins, and minerals [4-6]. Traditionally, the herb was used as a food source and for curing various diseases [7,8].

Every stage of the extraction process, from pre-extraction to the extraction itself, holds equal significance in the study of medicinal plants. The amount of the extracted compounds is significantly affected by the extraction method, temperature, solvent, time, and agitation speed. A diverse array of technologies employing various extraction methods is available today, categorized as conventional and modern techniques. Conventional methods involve the use of solvents and typically demand extended extraction times, whereas modern extraction methods have been increasingly utilized in the extraction of natural products [9]. Hence, it is necessary to compare the extraction yields obtained from the most commonly used methods and to evaluate the effect of solvent and time on the extracted amount of bioactive compounds [10,11].

This study aimed to optimize the extraction conditions of bioactive compounds from Kurdistan *U. dioica *by comparing the extraction yields obtained from conventional and modern extraction techniques by using different solvents for different extraction times.

## Materials and methods

Plant preparation

During the summer months of 2022, specifically between June and August, fresh plants were gathered from the northern region of Kurdistan, Iraq. Subsequently, the collected plants underwent a process that involved cleaning, washing with water, and shade drying at room temperature. Following this, the leaves and stems were ground and converted into powder using a blender. The resulting powders were carefully stored in clean plastic containers, ensuring protection from heat, moisture, light, and until their intended use. All the experiments were conducted in triplicate.

Maceration method

**Figure 1:**
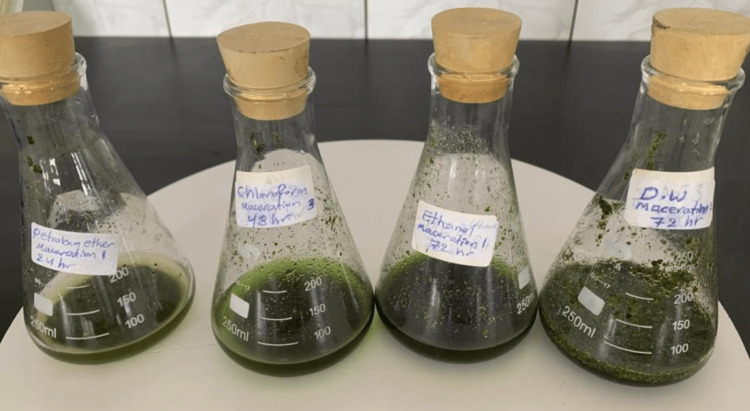
Maceration extraction of Urtica dioica leaves and stems using (petroleum ether, chloroform, ethanol, and distilled water) solvents.

Two grams of pulverized stems and leaves obtained from the *U. dioica* plant were combined with 50 mL of different solvents (Figure 1), namely, petroleum ether, chloroform, ethanol, and water (when compared to ethanol, methanol is considered a hazardous substance due to its high risk of causing poisoning, so it is avoided) in sealed conical flasks. These mixtures went through interaction for various durations (24, 48, and 72 hours) while consistently stirring at room temperature. Following this, the resulting extracts were collected and filtered. The samples were subsequently concentrated using a rotating evaporator at a temperature of 40°C under reduced pressure. Finally, the concentrated extracts were weighed and stored at a temperature of -20°C [12].

Soxhlet method

**Figure 2:**
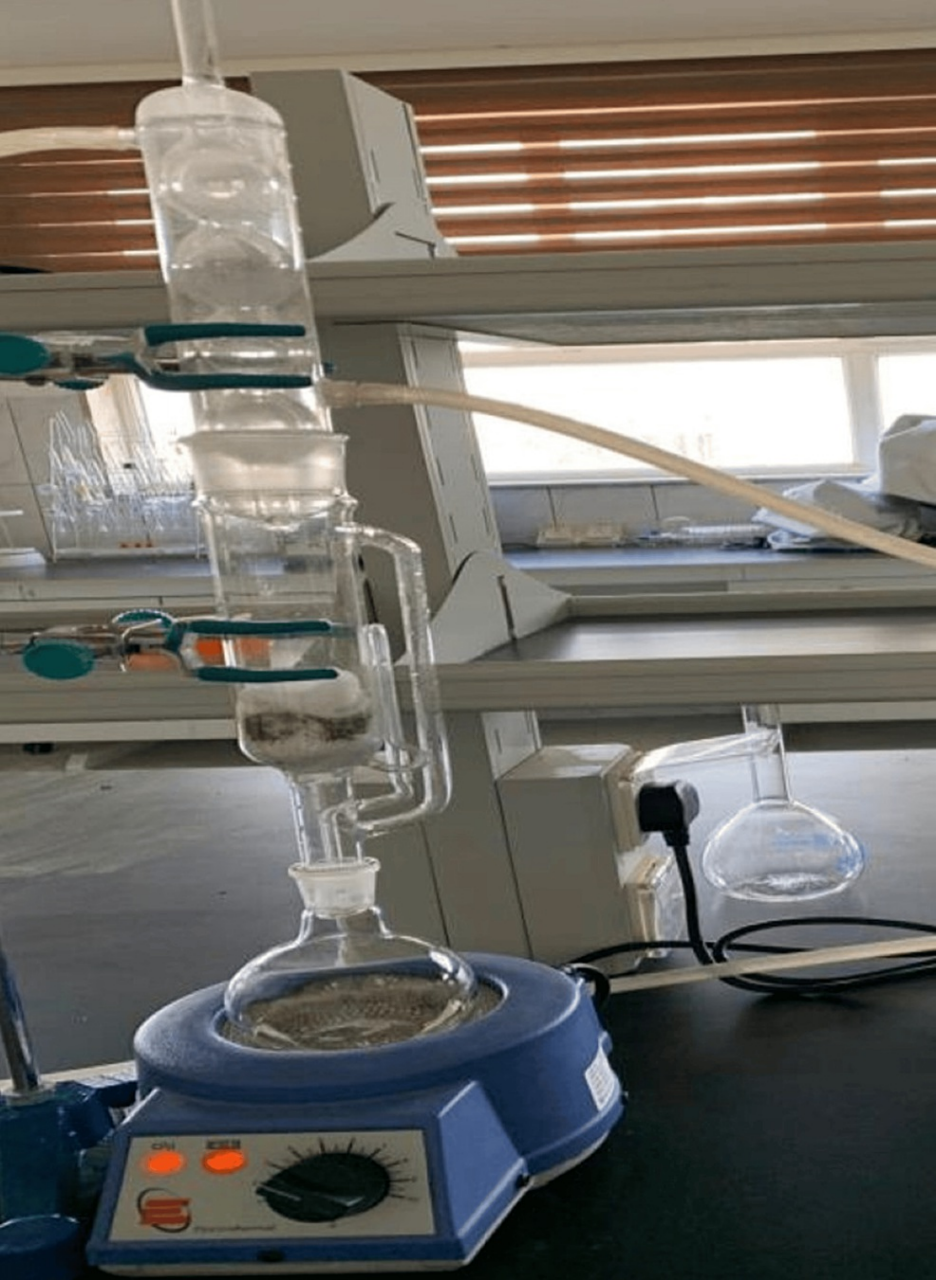
Soxhlet extraction of Urtica dioica leaves and stems using (petroleum ether, chloroform, ethanol, and distilled water) solvents.

Two grams of leaves and stems experienced Soxhlet extraction with a 50 mL solvent (including petroleum ether, chloroform, ethanol, and water) for durations of three, 12, and 24 hours. The obtained extracts underwent filtration (Figure 2), and the resulting filtrate was then evaporated under reduced pressure to acquire a crude extract [13].

Ultrasound-assisted extraction (UAE) method

**Figure 3:**
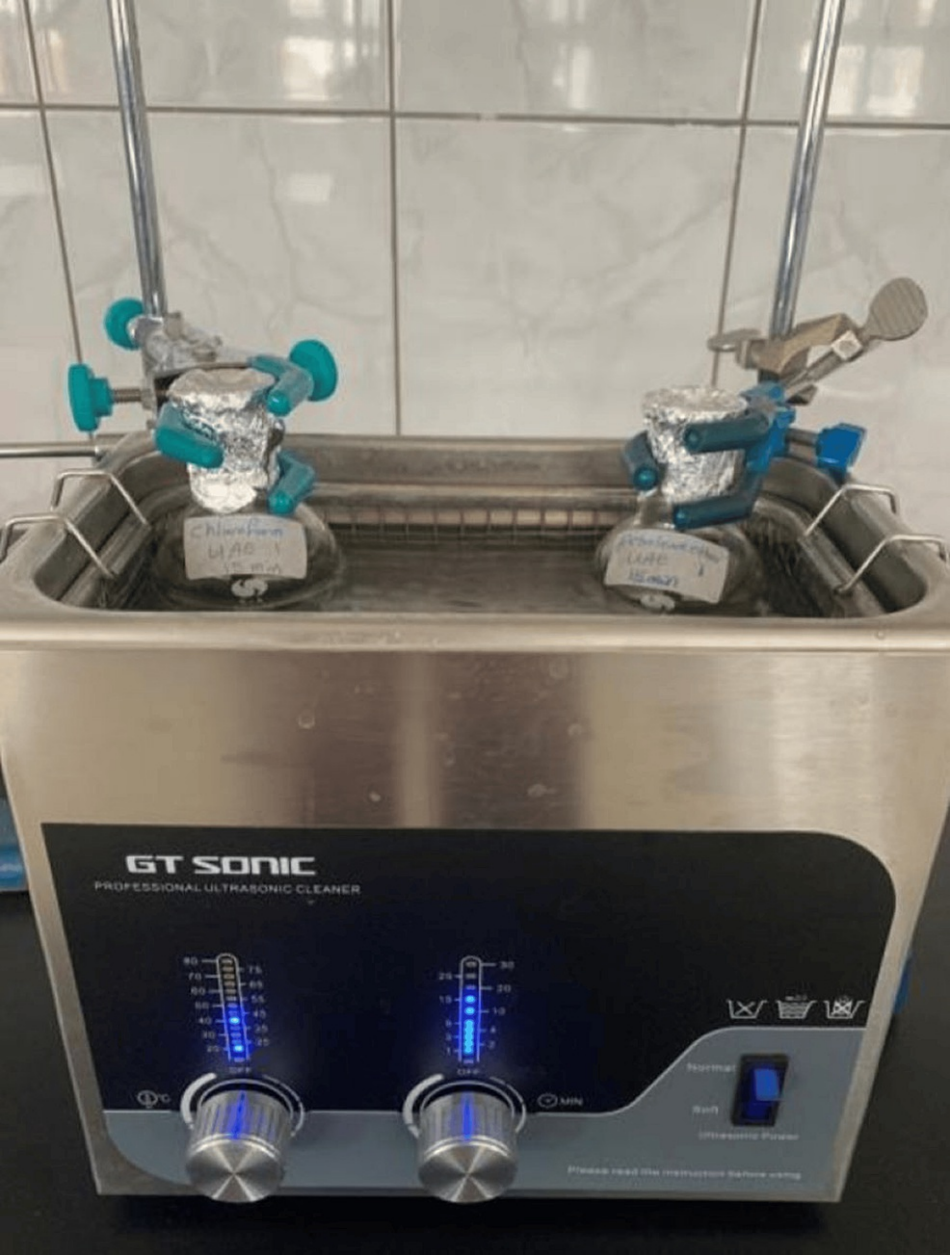
Ultrasound assisted extraction of Urtica dioica leaves and stems using (petroleum ether, chloroform, ethanol, and distilled water) solvents.

Two grams of finely ground stems and leaves were placed in a 100 mL flask, followed by the addition of 50 mL of solvent (petroleum ether, chloroform, ethanol, and water). UAE was conducted at 400 W and 40°C for durations of 15, 30, and 60 minutes (Figure 3) [14].

Microwave-assisted extraction (MAE) method

**Figure 4:**
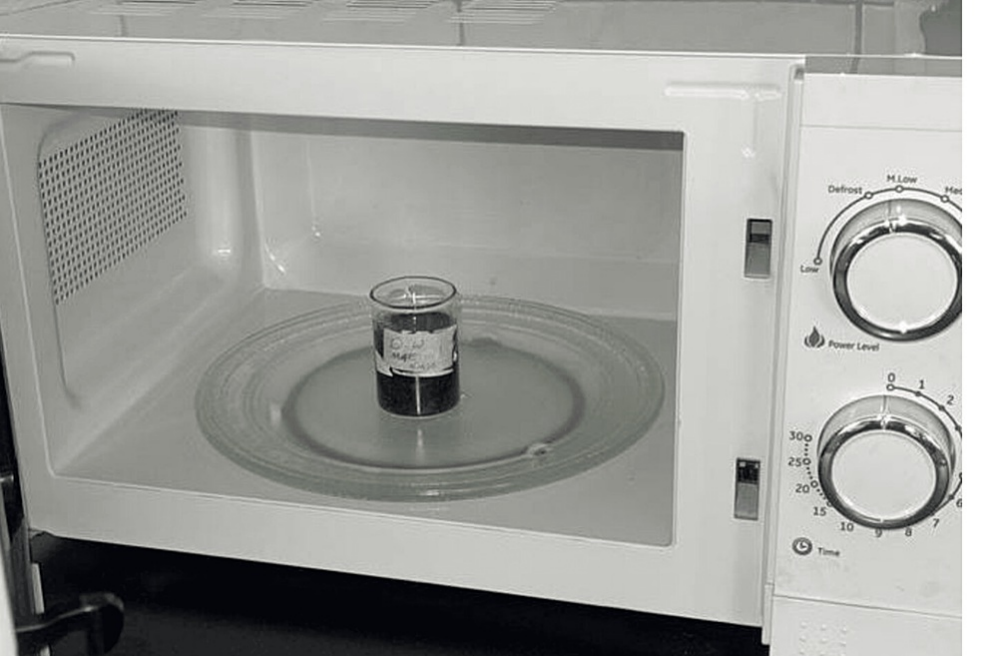
Microwave-assisted extraction of Urtica dioica leaves and stems using (petroleum ether, chloroform, ethanol, and distilled water) solvents.

A household microwave oven (MWO-261-01, Newal, China), having a total capacity of 700 W, 230-240 V ~ 50 Hz, was employed in this study. Plant samples (1 g) were combined with (25 mL) of the same solvents used in other methods in flat-bottomed beakers, which were then placed inside the microwave oven. The resulting mixtures underwent microwave irradiation (700 W power) following the approach outlined by Pan et al. [15] with minor adjustments to achieve irradiation times of two, four, and six minutes: 45 seconds power on, followed by 30 seconds power off, and then 15 seconds power on (Figure 4). Following each 60-second irradiation, the sample was permitted to cool down to room temperature. Before measuring the mass of the extracts, the samples were filtered, and a rotary evaporator was used to concentrate them at 40°C under lowered pressure [15,16].

**Figure 5:**
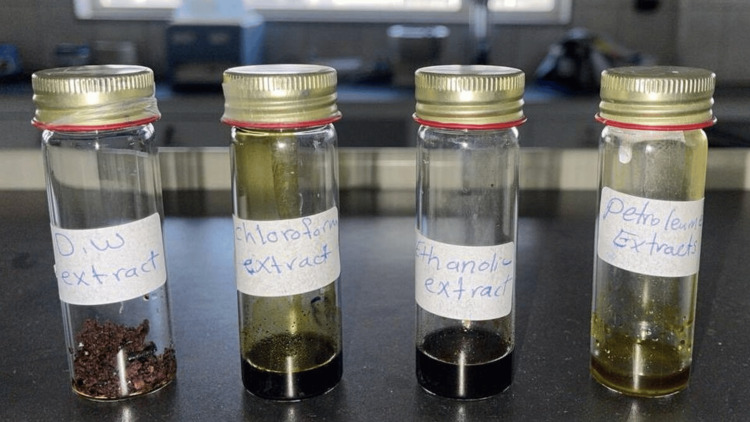
Urtica dioica leaves and stem extracts prepared by using (petroleum ether, chloroform, ethanol, and distilled water) solvents.

*U. dioica* leaves and stem extracts prepared by using four different solvents were used for extraction: petroleum ether, chloroform, ethanol, and distilled water. Each solvent was chosen for its specific polarity to target different classes of phytochemicals (Figure 5).

Statistical analysis

A one-way analysis of variance (ANOVA) test was applied to detect differences among four different extraction techniques in terms of extraction yield amount affected by solvent type and extraction time. Each test was conducted at least three times, and the data are presented as the mean ± standard deviation (SD). The data were calculated using Microsoft Excel and analyzed with Prism software (version 10.0.3; GraphPad, San Diego, CA). Differences were considered to be significant at p < 0.05.

## Results

To evaluate the optimal conditions for extraction of bioactive compounds from 2 g of *U. dioica *leaves and stem sample, four extraction techniques (maceration, Soxhlet, UAE, and MAE) were evaluated and compared by measuring the number of extraction yields produced by solvents, namely, petroleum ether, chloroform, ethanol, and water at different extraction times.

Maceration extraction yields

The total amount of extraction yields generated by the maceration technique by using solvents such as petroleum ether, chloroform, ethanol, and water was assessed over 24, 48, and 72 hours to identify the optimal conditions for the extraction of bioactive compounds. Table 1 provides a summary of the influence of solvent type and extraction time intervals on the amount of extraction yields (measured in milligrams) obtained through the maceration extraction method. As presented in Table 1, a definite correlation between the amount of extraction yield and increasing extraction time was noted, as none of the solvents produced the highest amount of extraction yields at 24 hours. Additionally, the solvent type showed a relevant effect on the amount of extraction yields. The highest extract yield produced by the non-polar solvent petroleum ether was 38.53 mg at 48 hours. Chloroform showed the highest yield at 72 hours (45.133 mg). Among organic solvents, ethanol significantly gave the highest amount of extracts (79.6 mg) at 72 hours. The highest yield gained from the most polar solvent water was 303.37 mg at 72 hours.

**Table 1:** Comparing the effects of different solvent types and extraction time points on the total extraction amount (mg) using the maceration extraction technique. Values are expressed as means ± SD of three measurements. Values with different letters differ significantly within each row, and values with different symbols differ significantly within each column (p < 0.05, one-way ANOVA). *Values are means ± SD of three measurements. EtOH: Ethanol, D.W: Distilled water

Solvent/Time (hr)	Petroleum ether	Chloroform	EtOH	D.W
24	35.40 ± 3.75 ^a^^ ¥^	44.43 ± 0.85 ^b ¥^	56.24 ± 1.35 ^c ¥^	277.20 ± 1.91 ^d ¥^
48	38.53 ± 3.26 ^a ¥^	43.07 ± 0.84 ^a ¥^	70.07 ± 1.92 ^b €^	267.63 ± 2.39 ^c €^
72	38.53 ± 0.47 ^a ¥^	45.13 ± 4.32 ^a ¥^	79.60 ± 2.46 ^b ‡^	303.37 ± 2.38 ^c ‡^

As presented in Table 1, chloroform showed the highest yield at 72 hours (45.133 mg). Among organic solvents, ethanol significantly gave the highest amount of extracts (79.6 mg) at 72 hours. The highest yield gained from the most polar solvent water was 303.37 mg at 72 hours.

Soxhlet extraction yield

Table 2 summarizes the mass of the extracts that were obtained (in milligrams) using the Soxhlet technique impacted by solvent type and extraction duration. The type of solvent used and the length of the extraction process had a significant impact on the amount of extractable matter. As indicated in Table 2, ethanol proved to be an organic solvent with the highest efficiency generating the highest amount of extracts, while the non-polar petroleum ether gave the lowest extraction yields. By extending the extraction time, all the solvents employed in the Soxhlet extraction process, namely, petroleum ether, chloroform, ethanol, and water, exhibited their highest yields at the 24-hour mark, resulting in amounts of 49.82 mg, 57.7 mg, 162.3 mg, and 674.26 mg, respectively.

**Table 2:** Comparing the effects of different solvent types and extraction time points on the total extraction amount (mg) using the Soxhlet extraction technique. Values are expressed as means ± SD of three measurements. Values with different letters differ significantly within each row, and values with different symbols differ significantly within each column (p < 0.05, one-way ANOVA). Values are means ± SD of three measurements. EtOH: Ethanol, D.W: Distilled water

Solvent/Time (hr)	Petroleum ether	Chloroform	EtOH	D.W
3	39.98 ± 1.22 ^a ¥^	54.30 ± 3.62 ^b ¥^	68.37 ± 2.37 ^c ¥^	286.93 ± 2.46 ^d ¥^
12	41.43 ± 0.21 ^a ¥^	52.35 ± 2.22 ^b ¥^	85.18 ± 1.57 ^c €^	466.40 ± 1.78 ^d €^
24	49.82 ± 2.05 ^a €^	57.70 ± 4.37 ^b ¥^	162.30 ± 2.80 ^c ‡^	674.26 ± 1.63 ^d ‡^

UAE yields

In UAE, the extraction yield was also affected by the solvent type and extraction duration as that of conventional methods. The significant impact of the type of solvent and extraction time on the amount of collected extracts was arranged in Table 3. In terms of solvent type, non-polar solvents, such as petroleum ether, yield lesser amounts of extracts compared to polar solvents such as water.

**Table 3:** Comparing the effects of different solvent types and extraction time points on the total extraction amount (mg) using the ultrasound-assisted extraction technique. Values are expressed as means ± SD of three measurements. Values with different letters differ significantly within each row, and values with different symbols differ significantly within each column (p < 0.05, one-way ANOVA). Values are means ± SD of three measurements. EtOH: Ethanol, D.W: Distilled water

Solvent/Time (min)	Petroleum ether	Chloroform	EtOH	D.W
15	38.77 ± 0.96 ^a ¥^	54.37 ± 2.43 ^b ¥^	64.70 ± 3.27 ^c ¥^	119.02 ± 0.40 ^d ¥^
30	45.73 ± 0.85 ^a ¥^	56.93 ± 2.47 ^b ¥^	78.77 ± 2.0 ^c €^	464.53 ± 2.64 ^d €^
60	55.37 ± 0.76 ^a ¥^	59.20 ± 2.07 ^a ¥^	89.07 ± 2.55 ^b ‡^	680.40 ± 1.37 ^c ‡^

The quantity of extracts acquired from all the solvents utilized in this process increased when the extraction time was extended from 15 and 30 minutes to 60 minutes. Petroleum ether produced the highest quantity of extracts at 60 min (55.37 mg). Table 3 indicates that, after 60 minutes, chloroform produced the highest yield of extracts (59.2 mg). The most effective organic solvent was ethanol, which resulted in elevated yields at 60 min (89.07 mg). Additionally, the polar solvent water offers the maximum extract yields after 60 min (680.4 mg).

MAE yield

The solvent type and extraction duration had an impact on extracted yields, much like in previous techniques. Table 4 shows the substantial effect of solvent type and duration of extraction on the quantity of extracts produced utilizing the microwave approach. Ethanol needed a shorter time (four min) to give the largest quantity of extract yields (178.3 mg). At six min, petroleum ether, chloroform, and water gave the highest yields (59.67 mg, 62.2 mg, and 802.67 mg, respectively). Petroleum ether, being non-polar, is not effective in MAE because of its low dielectric constant, resulting in the lowest yield. Conversely, polar solvents such as water, with a high dielectric constant, produce a significant amount of extracts.

**Table 4:** Comparing the effects of different solvent types and extraction time points on the total extraction amount (mg) using the microwave-assisted extraction technique. Values are expressed as means ± SD of three measurements. Values with different letters differ significantly within each row, and values with different symbols differ significantly within each column (p < 0.05, one-way ANOVA). Values are means ± SD of three measurements. EtOH: Ethanol, D.W: Distilled water

Solvent/Time (min)	Petroleum ether	Chloroform	EtOH	D.W
2	36.07 ± 0.93 ^a ¥^	57.07 ± 3.98 ^b ¥^	137.70 ± 1.32 ^c ¥^	157.10 ± 3.11 ^d ¥^
4	44.40 ± 0.62 ^a €^	58.47 ± 2.0 ^b ¥^	178.30 ± 1.67 ^c €^	534.37 ± 2.99 ^d €^
6	59.67 ± 1.46 ^a ‡^	62.20 ± 2.09 ^a ¥^	178.30 ± 1.31 ^b €^	802.67 ± 2.76 ^c ‡^

## Discussion

The investigation into the bioactive chemical extraction from *U. dioica* provides a thorough knowledge of the plant's diverse phytochemical composition and historical applications. Traditionally, the plant was used for managing cardiovascular issues such as hypertension, and it has also been found to help regulate glucose levels and may even have a protective effect against prostatic hyperplasia. Extracts from nettle leaf show promise as anti-inflammatory agents for conditions such as rheumatoid arthritis. Additionally, the extracts could enhance the effectiveness of breast cancer treatment by increasing the sensitivity of cancer cells to certain medications such as paclitaxel [7].

The study meticulously compares conventional approaches such as maceration and Soxhlet extraction, with modern approaches such as UAE and MAE. The yields of extracted matter were significantly influenced by the extraction techniques, the polarity of solvents, and extraction time. Thus, the efficiency of extracting bioactive components meaningfully relies on these factors [17,18]. Notably, the results underscore the efficiency of non-conventional methods in yielding bioactive compounds in shorter durations, reflecting a contemporary emphasis on sustainable and time-effective extraction processes. The findings bear direct implications for industries reliant on plant-derived compounds, emphasizing the need for tailored extraction methods based on the targeted bioactive compounds. Furthermore, the study encourages thoughtful consideration of the environmental footprint, promoting non-conventional methods as environmentally friendly alternatives due to their reduced time and solvent requirements.

The choice of solvents, encompassing non-polar (petroleum ether) to polar (water) varieties, significantly impacts the extraction yields, with ethanol consistently emerging as an effective solvent across diverse extraction methods. Usually, a variety of solvents, including alcohols, ether, acetone, and water, are used to extract bioactive substances from natural sources due to their broad solvent solubility tendency, with water being commonly utilized for extracting highly polar components such as amino acids, glycosides, and carbohydrates. On the other hand, low-polarity components such as aromatic compounds are extracted using ether.

Consequently, mixtures of alcohol and water are employed to extract a diverse array of ingredients with varying solubility characteristics. That is why different solvents, whether in pure form or as combinations, have been utilized in many studies to extract bioactive constituents with diverse polarities [19].

The utilization of water, in conjunction with various organic solvents, transforms it into a moderate polar environment, creating favorable circumstances for extraction. Additionally, when water is added to alcohols, plant materials expand more readily, increasing the surface area of interaction between the solvent and the plant matrix. This ultimately contributes to an improvement in the extraction yield. Several investigations verified that polar solvents inevitably produce greater amounts of phenolics in different plant species than non-polar solvents [20,21].

In the maceration method, the most effective organic solvent was ethanol and generated the most amount of extracts, bypassing methanol use due to its toxic effects. On the other side, water, as a highly polar solvent, and petroleum ether, as a non-polar solvent, are improper for extracting out high-level polar materials. Furthermore, using water as a solitary solvent for extraction produces yields containing various adulterants, besides polar compounds, and this interrupts the identification and quantification processes, as mentioned in the study of Chirinos et al. [22].

The extract yields of Soxhlet and UAE with the application of solvents (petroleum ether, chloroform, ethanol, and water) were higher than the yields of maceration extraction and lesser than the yields of MAE. These findings came in agreement with the work of Bandar et al. [11] and Drosou et al. [23]. The extraction yields of UAE were notably lower compared to those of MAE. It is recognized that the MAE process raises the temperature, thereby reducing the solvent viscosity. Consequently, the solvent becomes capable of dissolving the desired component by wetting the sample matrix [24,25]. Moreover, it induces localized heating within the moist sample, serving as a catalyst for breaking down the plant matrix. This facilitates the release of the solute, allowing it to diffuse and dissolve in the solvent [26]. Within the context of UAE, the solvent's improved penetration into cellular materials is a result of the mechanical effect of ultrasound. The breaking down of cell walls results in this enhancement in mass transfer. As for the length of the extraction process, the significant yields obtained in the first five minutes of the water-based UAE and MAE show that the destruction of cells improves the washing step. This increases mass transfer and penetration of a solvent [23]. It is worth mentioning that, MAE, the non-polar solvent could significantly produce lesser extract yields than polar solvents. This can be explained by the solvent's varying dielectric characteristics. While polar solvents, such as ethanol, have a strong microwave absorption capacity and thus heat up faster and can improve the extraction process, non-polar solvents are transparent to microwave radiation and do not heat up under the microwave. Therefore, a key factor in microwave extraction is the solvent's dielectric characteristics towards microwave heating. This explains why extract yields from ethanol and water were far higher than those from petroleum ether, which yielded very little. Water showed better yields during the whole extraction process because the dielectric constant of water is higher than that of ethanol. The primary mechanism to increase the yield in the MAE process was the dipole rotation of the polar solvent in the microwave field. The target chemical may swiftly enter the extraction solution and absorb sufficient amounts of microwave radiation. Ethanol is used over water because of its superior heating efficiency and greater capacity to solve bioactive chemicals. This was clearly explained in the review of Mandal et al. [27].

A significant relationship was found between the extraction yield and longer extraction times. As the maceration extraction duration extends (from 24 hours to 72 hours), the extraction yield using different solvents (chloroform, ethanol, and water) increases. However, in the case of petroleum ether, the extraction yield rises from 24 hours to 48 hours and then stabilizes, remaining constant thereafter. The conclusion drawn is that the best time for extraction varies depending on the solvent used. This aligns with what Fick's second law of diffusion explains, a state of final equilibrium is reached after a specific time between the concentrations of solute in the plant matrix and the bulk solution (solvent). This implies that an extended extraction time is not beneficial for extracting additional compounds. Prolonged extraction processes may even result in oxidation due to exposure to light or oxygen [28]. As shown in Tables 2-3, there was a clear relationship between the increase in time and the extraction yield obtained from Soxhlet and UAE, respectively. Table 4 demonstrates a definite relationship between the increase in time and the extraction yield. As the duration extends from two minutes to six minutes, the extracted products show an increase when various solvents (petroleum ether, chloroform, and water) are used. However, when ethanol is employed, the maximum extraction yield is achieved within a shorter time (four minutes), after which it remains constant at 178.3 mg as the time extends to six minutes. Extended exposures give greater extraction yields; however, increasing the irradiation period further does not improve extraction performance and can even cause a decrease in concentration yield. There is always a chance that prolonged exposure will cause degradation from heating. These findings came in accordance with the works of Bandar et al. [11] and Rafiee et al. [28].

The primary limitations of this study stem from several factors: the presence of humidity during the shade drying phase of plant samples in summer, the purity of the solvents utilized, and the lack of advanced ultrasound and microwave instruments. By addressing these identified limitations, it is anticipated that a higher quantity of extraction yields could be achieved.

## Conclusions

Extraction is a very critical step in the study of medicinal plants. The amount of extracted compounds is significantly affected by the extraction method, solvent, and time. Concerning the solvents applied, ethanol proves to be the most effective solvent used in various extraction techniques, giving the highest extractive matter of bioactive compounds, and petroleum ether produced the lowest yields. Additionally, extraction yield shows a direct relation with extraction time. Among conventional methods, Soxhlet was the most powerful method and had the most extraction yields, while maceration had the lowest yields. The modern techniques surpassed the conventional methods by producing high extraction yields within a shorter time (a few minutes) and using a lesser amount of solvent. Consequently, UAE and MAE emerge as the most efficient techniques. Hence, MAE offers numerous benefits in comparison to alternative methods. These advantages stem from its shortened extraction time, enhanced efficiency in extraction, reduced labor requirements, and heightened selectivity in extracting bioactive compounds such as phenolics, anthraquinone, and coumarins. Thus, it emerges as a preferred method for bioactive compound extraction from aerial parts of *U. dioica.*
